# Integrated Physiologic and Proteomic Analyses Reveal the Molecular Mechanism of *Navicula* sp. in Response to Ultraviolet Irradiation Stress

**DOI:** 10.3390/ijms25052747

**Published:** 2024-02-27

**Authors:** Siyu Gong, Pan Pan, Xiangying Meng, Yuxin Zhang, Hanli Xu, Honggang Hu, Xiyu Cheng, Qiong Yan

**Affiliations:** 1 College of Life Sciences and Bioengineering, School of Physical Science and Engineering, Beijing Jiaotong University, Beijing 100044, China; 21272004@bjtu.edu.cn (S.G.); panpan3366@mail.tust.edu.cn (P.P.); x4meng@uwaterloo.ca (X.M.); xuhanli@bjtu.edu.cn (H.X.); hghu@bjtu.edu.cn (H.H.); 2 College of Materials Science and Engineering, Chongqing University, Chongqing 400045, China; zhangyuxin@cqu.edu.cn

**Keywords:** *Navicula* sp., ultraviolet irradiation, chlorophyll, antioxidant system, proteomics

## Abstract

With the continuous development of space station construction, space ecosystem research has attracted increasing attention. However, the complicated responses of different candidate plants and algae to radiation stress remain unclear. The present study, using integrated physiologic and proteomic analyses, was carried out to reveal the molecular mechanism of *Navicula* sp. in response to ultraviolet (UV) irradiation stress. Under 12~24 h of high-dose UV irradiation conditions, the contents of chlorophyll and soluble proteins in *Navicula* sp. cells were significantly higher than those in the control and 4~8 h of low-dose UV irradiation groups. The activity of catalase (CAT) increased with the extension of irradiation time, and the activity of superoxide dismutase (SOD) decreased first and then increased. Furthermore, differential volcano plot analysis of the proteomic data of *Navicula* sp. samples found only one protein with a significant difference. Differential protein GO analysis unveiled that UV irradiation can activate the antioxidant system of *Navicula* sp. and further impact photosynthesis by affecting the photoreaction and chlorophyll synthesis of *Navicula* sp. The most significant differences in KEGG pathway analysis were also associated with photosynthesis. The above results indicate that *Navicula* sp. has good UV radiation resistance ability by regulating its photosynthetic pigment content, photosynthetic activity, and antioxidant system, making it a potential candidate for the future development of space ecosystems.

## 1. Introduction

Diatoms, which have played an important role in ecosystems as primary producers, are attracting increasing attention [1]. They have made significant contributions to the early development of life on Earth. The siliceous shells of diatoms are widely used as a bioactive substance and biomass energy in industries such as environmental monitoring, energy development, metallurgy, chemical industry, electric power, agriculture, fertilizers, and building materials [2]. *Navicula* sp. is a benthic diatom that is not only an ideal opening bait for valuable aquatic animals such as the abalone and sea cucumber, but also a marine organism with potential pharmaceutical value and a potential oil-rich microalgae, hence deserving further research and development.

With the rapid development of space station construction and deep space exploration, the focus has shifted from exploiting diatoms on Earth to exploring their potential in the vastness of space [3,4,5]. Environmental control and life support systems (ECLSSs) are essential for human survival in the harsh environment of space. Biological systems can be incorporated into spacecrafts to fundamentally mimic the balance of photosynthesis and respiration in the Earth’s ecosystem, with an increased reuse of biomass throughout the food chain [4,6]. Algae (e.g., *Navicula* sp.), which can survive even after prolonged exposure to certain harsh conditions (e.g., irradiation, vacuum, and extreme temperature), are considered potential candidates for the development of future ECLSSs. Therefore, it is of great importance to investigate their responses to different stress conditions [3].

UV irradiation is short-wave radiation in space; it is destructive to life, and it is of significant importance in biology. The effects of UV irradiation on diatoms are mainly demonstrated in changes in cellular structure and metabolic functions, including damage to chloroplast membranes, changes in photosynthetic pigment content, decreased photosynthetic efficiency, and alterations in antioxidant systems [7,8,9,10,11]. These changes significantly affect diatom growth and physiological metabolism [12]. Many researchers have studied the effects of UV irradiation on algae, mostly focusing on the impact of UV-B radiation on microalgae. Some studies about the effect of UV irradiation on diatom proteins mainly focus on protein content, and some results are inconsistent. Some studies indicate that UV irradiation disrupts the normal physiological activities of algae and inhibits protein synthesis and reduces protein content [13,14,15,16,17,18,19]. However, some other studies claim that certain doses of UV irradiation can enhance protein synthesis and increase protein content [15]. Algal chlorophyll content decreases under stronger UV irradiation and chlorophyll a (Chla) is more susceptible to damage than chlorophyll b (Chlb) [13,14,15,16,17]. Besides affecting photosynthetic pigments, UV irradiation can also influence the metabolic processes of other plant pigments, particularly the synthesis of ultraviolet barrier pigments [18,19]. Analyses of differentially expressed proteins (DEPs) could provide a molecular-level explanation for the physiological response of algae to environmental stress. However, there are relatively few studies on the radiation response of *Navicula* sp. based on proteomics, and its regulatory metabolic networks in response to radiation stress remain unclear.

In this work, integrated physiologic and proteomic analyses were carried out to provide a comprehensive overview of the molecular mechanism of irradiation stress response in *Navicula* sp., a promising candidate for space ecosystems [6,20]. Firstly, chlorophyll and soluble protein levels as well as the expression of antioxidative enzymes were recorded to investigate the physiologic responses of *Navicula* sp. under ultraviolet irradiation. Furthermore, proteomic analysis was performed on *Navicula* sp. before and after irradiation to identify DEPs in this response process. Afterwards, the metabolic responses of *Navicula* sp. under UV irradiation were illustrated using GO and KEGG analysis. The findings of this study present a better understanding of the molecular mechanism underlying the irradiation stress response in *Navicula* sp., hence providing valuable information to the future development of space ecosystem.

## 2. Results

### 2.1. Effect of UV Irradiation Dose on Chlorophyll Content in Navicula sp.

**Figure 1:**
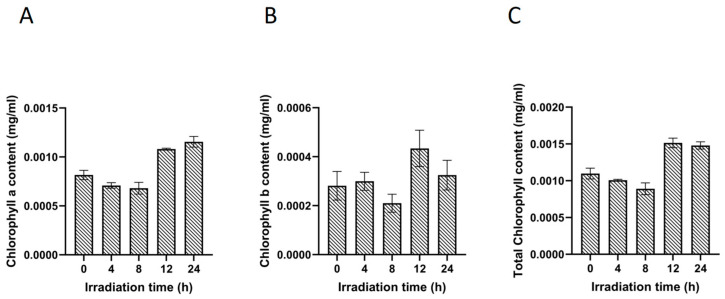
Effect of different doses of ultraviolet light on chlorophyll content of *Navicula* sp. (**A**) Effects on chlorophyll a; (**B**) effects on chlorophyll b; (**C**) effects on total chlorophyll.

The changes in the relative chlorophyll content of *Navicula* sp. after UV irradiation treatment are shown in Figure 1. Compared to the control group, under 4~8 h of low-dose UV radiation conditions, there was only a slight change in chlorophyll a, chlorophyll b, and total chlorophyll contents (i.e., total chlorophyll content, 0.00089~0.00101 mg/mL vs. 0.00110 mg/mL) (Figure 1). Under 12~24 h of high-dose UV irradiation conditions, the contents of the chlorophyll a, chlorophyll b, and total chlorophyll in *Navicula* sp. cells were significantly higher than those in the control and 4~8 h of low-dose UV irradiation groups (i.e., total chlorophyll content, 0.00148~0.00151 mg/mL vs. 0.00089~0.00110 mg/mL).

### 2.2. Effect of UV Irradiation Dose on Soluble Protein Content

**Figure 2:**
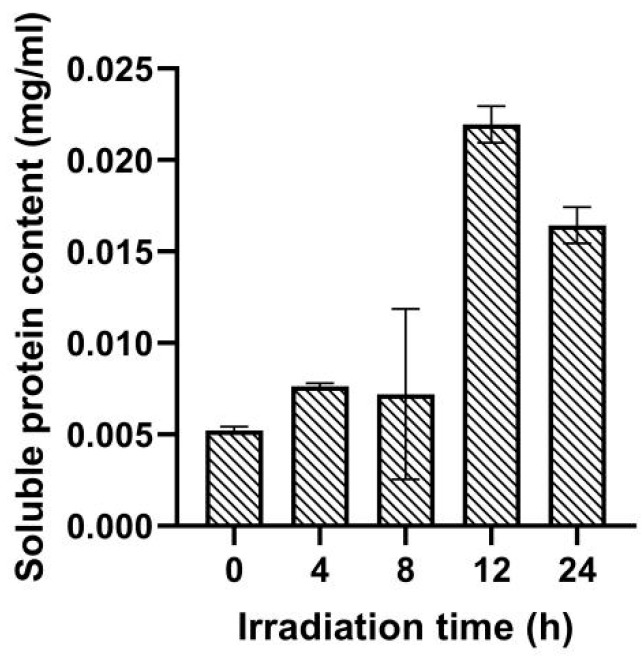
Effect of different doses of ultraviolet light on soluble protein content in *Navicula* sp.

The content of soluble protein in *Navicula* sp. after UV irradiation is shown in Figure 2. Compared to the control group, under 4~8 h of low-dose UV radiation conditions, a slight change in soluble protein content was recorded (0.0072~0.0076 mg/mL vs. 0.0052 mg/mL) (Figure 2). Under 12~24 h of high-dose UV irradiation conditions, soluble protein content in *Navicula* sp. cells was significantly higher than that in the control and 4~8 h of low-dose UV irradiation groups (0.016~0.021 mg/mL vs. 0.0052~0.0076 mg/mL). Intracellular soluble protein content increased with increasing irradiation time and showed obvious fluctuations. It rose sharply to 0.021 mg/mL in the case of 12 h of UV radiation and decreased slightly to 0.016 mg/mL in the 24 h of UV radiation group.

### 2.3. Effect of UV Irradiation Dose on CAT Activity

**Figure 3:**
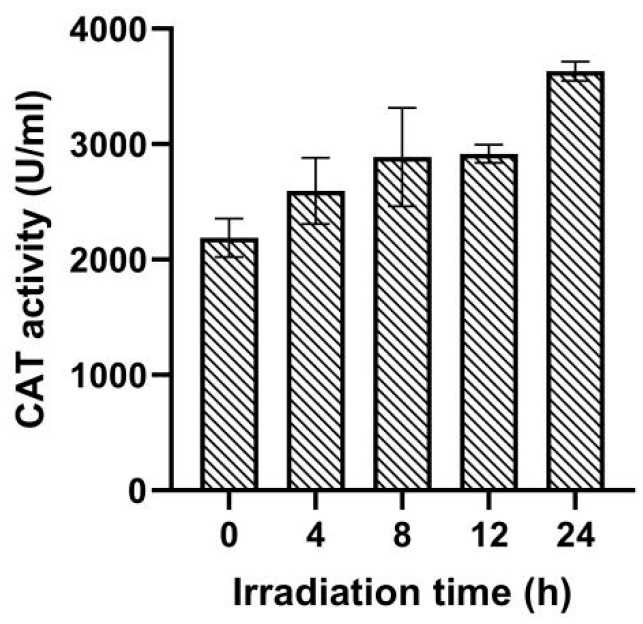
Effect of different doses of ultraviolet light on catalase activity in *Navicula* sp.

Reactive oxygen free radicals are closely associated with plant stress resistance. In a high-intensity radiation environment, more free radicals are produced in *Navicula* sp. cells, requiring more active antioxidant enzymes to remove them. The CAT activity of *Navicula* sp. cells after UV irradiation is shown in Figure 3. Under 4~24 h of UV radiation conditions, CAT activity increased with prolonged UV irradiation. Under 4~12 h of UV radiation conditions, CAT activity reached 2596~2917 U/mL, against 2188 U/mL observed in the control group (Figure 3). After 24 h of irradiation, CAT activity further increased to 3633 U/mL, an approximate increase of 66%.

### 2.4. Effect of UV Irradiation Dose on SOD Activity

**Figure 4:**
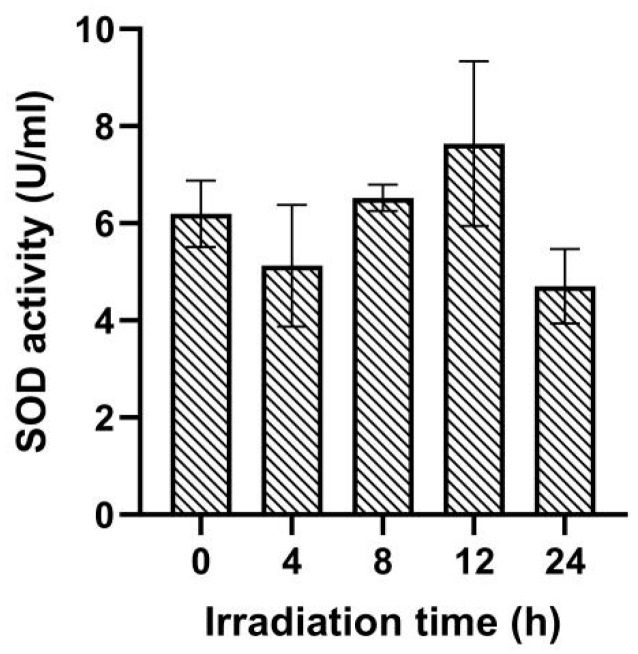
Effect of different doses of ultraviolet light on superoxide dismutase activity of *Navicula* sp.

As shown in Figure 4, after UV irradiation, the activity of SOD in *Navicula* sp. displayed a trend of initial decrease followed by an increase and then a subsequent decrease with the extension of irradiation time. SOD activity was 6.19 U/mL at 0 h. It decreased to 5.13 U/mL at 4 h, increased to 7.64 U/mL at 12 h, and then decreased sharply to 4.7 U/mL at 24 h.

### 2.5. Effect of UV Irradiation on Proteomics

#### 2.5.1. Differentially Expressed Proteins

**Figure 5:**
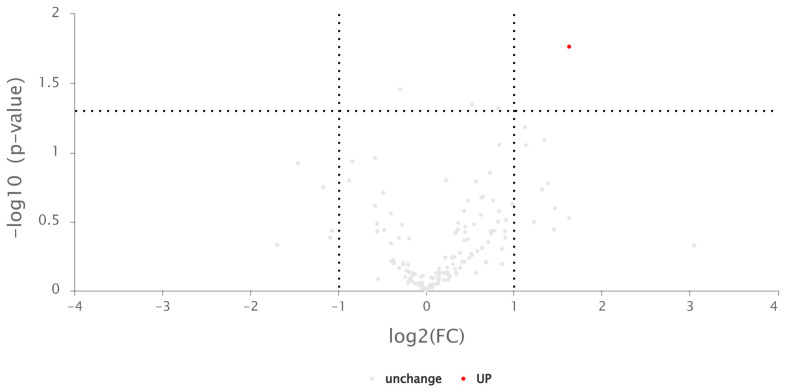
Volcanic maps of differential proteins between irradiated and unirradiated *Navicula* sp. The horizontal axis represents the fold change of differential proteins (log2 value), and the vertical axis represents the *p* value (−log10 value). Grey indicates proteins with no significant difference, and red represents upregulated proteins.

The mass spectrum data obtained from three repeated biological samples represent the whole proteome of the plants. In this sequencing, a total of 975,990 secondary spectra were obtained, including 5071 effective spectra. A total of 555 peptides and 189 proteins were identified. One identified protein whose content significantly increased after ultraviolet irradiation, namely α/β hydrolase, was screened. A differential volcano plot is shown below (Figure 5).

#### 2.5.2. Differential Proteins in GO Analysis

**Figure 6:**
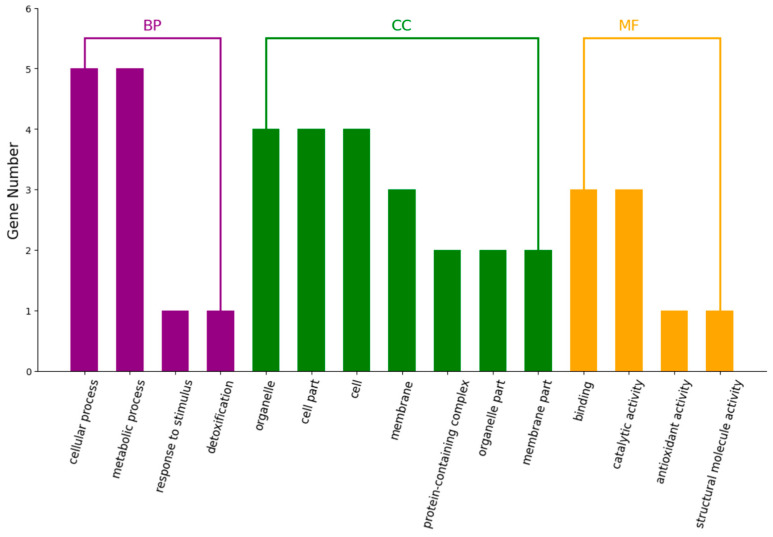
GO enrichment bar chart. The horizontal coordinate represents GO Level2 function annotation information. The purple group is biological processes, the green group is cellular components, and the orange group is molecular functions. The vertical coordinate represents the number of differentially expressed proteins under each functional classification.

Blast2Go 6.0 software was used to perform the GO functional annotation of all differentially expressed proteins. At the same time, the number of differential proteins was statistically analyzed at the GO secondary functional annotation level. According to functional characteristics, these differential proteins were divided into three categories: biological processes, cellular components, and molecular functions (Figure 6).

Proteins play an important role in *Navicula* sp.’s resistance to UV radiation. For biological processes, differential proteins in *Navicula* sp. after UV irradiation were mainly concentrated in cellular processes and metabolic processes. These related processes are mainly associated with oxidative stress. For cellular components, differential proteins in *Navicula* sp. were mainly concentrated in the organelles (cell parts) and in entire cells. In the case of molecular functions, they were mainly binding and catalytic activity (Figure 6).

#### 2.5.3. Differential Protein KEGG Pathway Analysis

**Figure 7:**
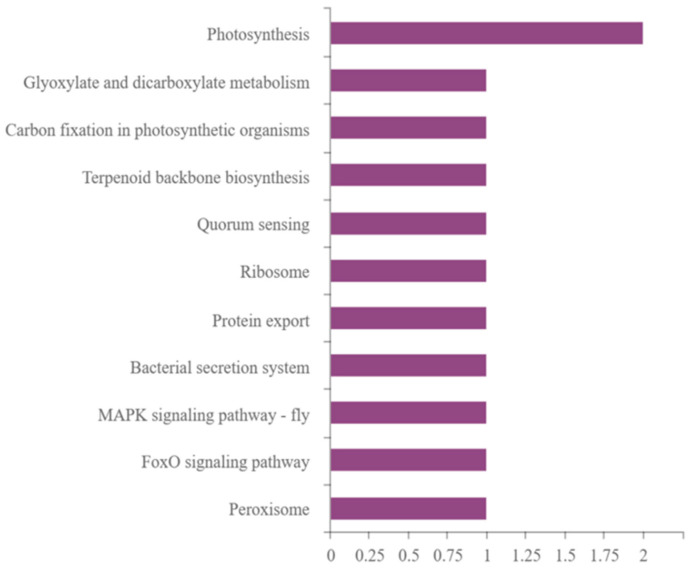
Column diagram of KEGG enrichment.

Using KAAS software (https://www.genome.jp/tools/kaas/, accessed on 3 April 2023), KEGG pathway annotation was performed on the set of differentially expressed proteins and the results are shown in Figure 7.

*Navicula* sp. has strong UV resistance with only a few differentially expressed proteins. Among them, the photosynthesis pathway had the most differentially expressed proteins, i.e., the pathway which was most affected by UV irradiation was the photosynthesis system. At the same time, the expression of some photosynthesis-related proteins decreased, and the expression of other proteins increased, indicating that the effect of UV irradiation on photosynthesis is not only inhibition. The proteins with increased content were dicarboxylic acid metabolism and peroxisome-related proteins. The proteins with decreased content included ribosomal proteins.

## 3. Discussion

UV irradiation, as an abiotic stress factor, can significantly influence various aspects of cellular growth and metabolism. Plants and algae, when subjected to stress, can self-adjust to counteract the adversity posed by UV irradiation stress. The present study was carried out to reveal the molecular mechanism of *Navicula* sp. in response to UV irradiation stress based on integrated physiologic and proteomic analyses.

The synthesis of chlorophyll plays an important role in the UV radiation response process. Under low-dose ultraviolet radiation conditions, there is only a slight change in chlorophyll content. Under 12~24 h of UV irradiation, the contents of both chlorophyll a and b significantly increased (Figure 1). Chlorophyll a is responsible for absorbing and transmitting light energy. The reduction in chlorophyll content under short durations of UV radiation could be attributed to varying degrees of damage caused by UV irradiation to the photosynthetic system and chlorophyll lamella structure within *Navicula* sp. cells. These changes led to dysregulation of photosynthetic pigments and impaired photosynthesis, decreased efficiency of light energy utilization, and diminished chlorophyll synthesis and repair capabilities [21,22]. UV irradiation may damage the phototransduction chain and weaken the ability of *Navicula* sp. cells to capture and transmit light energy. When exposed to longer periods with higher doses of UV irradiation, *Navicula* sp. cells may enhance chlorophyll synthesis through metabolic network regulation to withstand UV irradiation pressure.

During the UV radiation response process, the regulation of diverse protein synthesis plays an important role. Under 12~24 h of high-dose UV irradiation conditions, the soluble protein contents in *Navicula* sp. cells were significantly higher than that in the control group (Figure 2). Aromatic amino acids are precursors in the synthesis of flavonoids, and flavonoids have the ability to absorb UV radiation and protect plants from partial damage under UV exposure [23,24]. The increase in protein content in *Navicula* sp. could be partly attributed to the enhanced synthesis of aromatic amino acids. UV stress could activate the antioxidation system in algae, leading to the expression of more proteins to mitigate the cellular damage caused by UV irradiation. The longer the irradiation time lasted, the higher the CAT activity, while the dynamic value of SOD showed a trend of first increasing and then decreasing. Research on a wide range of algae indicates that UV irradiation can disrupt algal cellular physiological processes to varying degrees, leading to oxidative stress responses in cells [25]. UV irradiation stimulates the production of reactive oxygen species (ROS) in *Navicula* sp. In response, the *Navicula* sp. antioxidation system (e.g., CAT and SOD) is activated to scavenge these ROS, thereby protecting the cells and maintaining the photosynthetic activity of the algal cells [17]. However, as the intensity of UV irradiation increases, excessive UV light leads to damage to the molecular structure of SOD, and as a result, the induction of SOD vitality can be weakened. Additionally, UV irradiation may induce more responses from other antioxidant systems besides CAT and SOD. It was found that *Synechococcus* sp. PCC7942, a UV-B cyanobacteria, can rapidly synthesize anti-UV-B proteins [26].

Proteomic analysis further elucidates the response of *Navicula* sp. to UV irradiation stress. *Navicula* sp. shows high adaptability to harsh environments, as evidenced by the presence of only one significantly differentially expressed protein (i.e., α/β hydrolase) after 24 h of UV irradiation (Figure 5). α/β hydrolases typically engage in fundamental cellular metabolic processes, such as the breakdown and recycling of cellular metabolites, processing of external nutrients, and detoxification of xenobiotics. They play a pivotal role in metabolic regulation [27]. GO analysis of differentially expressed proteins indicates that UV irradiation could activate the antioxidation system of *Navicula* sp., which is consistent with experimental data. UV irradiation has the most significant impact on proteins related to biological processes in *Navicula* sp. Combining GO and KEGG analysis, it can be concluded that the antioxidation system, and proteins related to the light reactions and chlorophyll synthesis in the photosynthetic system, are most affected by UV irradiation.

When *Navicula* sp. cells are exposed to UV irradiation, it can trigger oxidative stress and disrupt normal physiological activities and protein synthesis. Specifically, this leads to the generation of large amounts of ROS, which can damage the cellular structure and function of algal cells [26,27,28,29]. In order to counteract these deleterious effects, algal cells have evolved a complex antioxidant defense system consisting of antioxidant enzymes and non-enzymatic antioxidants [30,31]. In antioxidant defense, most differential proteins are implicated in a number of biological processes, including the cellular response to superoxide, neutralization of oxygen radicals, cellular detoxification, reactive oxygen metabolism, the action of oxidative detoxifiers, and response to oxidative stress. The activation of these defensive processes is not only a response of algal cells to the toxicity of UV irradiation and part of their stress response strategies, but is also involved in the regulation of reactive oxygen species signaling [32]. After UV irradiation, free radical scavenging systems within *Navicula* sp. cells, such as peroxidases, can actively eliminate these free radicals through redox reactions to establish an effective defense mechanism [30,31,32,33]. These antioxidative responses and defense mechanisms within *Navicula* sp. cells constitute a complex biological process, a key strategy of algae like *Navicula* sp. against UV radiation.

The differential proteins of cellular components include those found in chloroplast thylakoids, Photosystem II, complex catalysts, and redox enzyme complexes. Chloroplast thylakoids are the internal structure of chloroplasts, containing the chlorophyll and proteins necessary for photosynthesis [34]. The differential expression of thylakoid proteins indicates the impact of UV on the photosynthesis of *Navicula* sp. The core complex of Photosystem II, which is crucial for photosynthesis, uses energy absorbed from light to split water and transfers the released electrons to plastoquinone [35,36]. UV light affects the photosynthetic rate of algal cells by impacting the light reactions in photosynthesis. This further confirms that Photosystem II is the most sensitive to UV radiation and suffers the most severe photoinhibition [37,38]. The cytochrome b6/f complex plays a central role in oxygen photosynthesis, linking electron transfer between photosystems I and II and converting light energy into a transmembrane proton gradient for ATP synthesis [39]. The B6/f complex is an intrinsic protein of the stroma-thylakoid membrane. The photoreactions of photosynthesis take place in the chloroplast’s thylakoid membrane, further suggesting that UV radiation affects photoreactions in photosynthesis in algal cells, which in turn affects the algal photosynthetic rate. Magnesium is an important element in the formation of chlorophyll structure. If magnesium ions cannot be bound, the porphyrin ring and its surrounding organic chain cannot be assembled, and so chlorophyll molecules cannot be formed [40,41]. The differential expression of magnesium ion-binding proteins in algal cells indicates that UV radiation affects the synthesis of photosynthetic pigments by influencing the magnesium ion-binding function of algal cells. The effects of UV radiation on photosynthesis are mainly reflected in the photoreaction and synthesis of photosynthetic pigments.

In summary, the antioxidation system and proteins related to light reactions and chlorophyll synthesis in the photosynthetic system are most affected in *Navicula* sp. under UV irradiation. It should be noted that 30% of the world’s animal protein comes from the ocean, and many algae are primary producers in aquatic ecosystems. Considering the excellent photosynthetic carbon sequestration capacity and stress tolerance of these algae (e.g., *Navicula* sp.), they may play an equally important role in future aerospace life support systems.

## 4. Materials and Methods

### 4.1. Materials

The experimental alga was purchased from the Freshwater Algae Culture Collection of the Chinese Academy of Sciences (Catalog No.: FACHB-1950), species name: *Navicula* sp.

The culture medium was CSI culture medium (composition detailed in Table 1) acquired from Freshwater Algae Culture Collection at the Institute of Hydrobiology (FACHB), National Aquatic Biological Resource Center (Wuhan, China).

The main reagents were as follows: chlorophyll assay kit; soluble protein assay kit; catalase (CAT) assay kit; superoxide dismutase (SOD) assay kit. All were purchased from the Nanjing Jiancheng Bioengineering Institute (Nanjing, China).

Method of soil extract preparation: A total of 200 g of garden soil which had not been fertilized was placed in a beaker or Erlenmeyer flask (Product number: I015452, Sichuan Shubo (Group) Co., Ltd, Sichuan, China). Then, 1000 mL of distilled water was added. The flask was sealed with an air-permeable stopper and heated in a water bath at boiling temperature for 3 h. After heating, it was allowed to cool and settled for 24 h. This process was repeated 3 times. Then, the mixture was filtered and the supernatant was collected. The clear liquid was sterilized in an autoclave and stored in a refrigerator at 4 °C for future use.

### 4.2. Experimental Methods

#### 4.2.1. Pre-Treatment before Irradiation

*Navicula* sp., which was in the logarithmic growth phase, was inoculated at a specified seeding density. The absorbance of the algae solution was measured every two days. After 8 days of incubation in an Erlenmeyer flask, the UV irradiation treatment was performed.

#### 4.2.2. Irradiation Treatment

A 300 VA ultra-clean workbench UV lamp tube (Harbin Donglian Electronic Technology Development Co., Ltd, Beijing, China) was used as the radiation source. The Erlenmeyer flasks were placed 20 cm below the UV lamp. Treatment durations were set at 4 h, 8 h, 12 h, and 24 h, with each duration having three independent repetitions.

#### 4.2.3. Treatment and Analysis after Irradiation Process

After irradiation, *Navicula* sp. samples continued to be cultured under standard conditions. Cultivation was carried out in 250 mL Erlenmeyer flasks, each containing 40 mL of CSI medium. The mouths of the flasks were sealed with a breathable but water-impermeable sealing film. The flasks were then placed under normal lighting conditions at room temperature, with a light/dark cycle of 12/12. The samples were harvested 96 h post-irradiation. After centrifugation at 3500 rpm for 10 min, the supernatant was reduced to 6 mL. The remaining *Navicula* sp. liquid was mixed and the cells were broken to obtain the homogenate. Subsequently, various physiological and biochemical indicators were measured, including the contents of chlorophyll a, chlorophyll b, total chlorophyll, soluble protein, and the activities of CAT and SOD. Additionally, proteomic analysis was conducted.

### 4.3. Assay Methods

#### 4.3.1. Chlorophyll a, Chlorophyll b, and Total Chlorophyll Content

First, 0.1 mL of homogenate was added to 1 mL of distilled water. This was mixed and the mixture was transferred to a 5 mL centrifuge tube. Then, the extraction solution was prepared (anhydrous ethanol/acetone = 1/2, *v*/*v*) and made up to 5 mL. The extraction solution was used for the extraction process in dark conditions for over 3 h until the solution turned completely white. After centrifuging at 3000 rpm for 10 min, the supernatant was diluted with the extraction solution to an appropriate concentration with an OD value of less than 1. The spectrophotometer with the extraction solution was zeroed and absorbance values were recorded at 645 nm and 663 nm. The following formulas were used for calculation:

Chlorophyll a content (mg/mL) = (12.7 × A_663_ − 2.69 × A_645_) × V × N/(v × 1000);

Chlorophyll b content (mg/mL) = (22.9 × A_645_ − 4.68 × A_663_) × V × N/(v × 1000);

Total chlorophyll content (mg/mL) = (20.2 × A_645_ + 8.02 × A_663_) × V × N/(v × 1000).

Note: V is the volume of absolute alcohol (5 mL);

N is the colorimeter determination multiple;

v is the volume of the sample (mL).

#### 4.3.2. Soluble Protein Content

Soluble protein content was determined using the Coomassie brilliant blue method. The homogenate was centrifuged at 3500 rpm for 10 min and the supernatant was collected. For the experimental group, 0.05 mL of supernatant was added to 3.0 mL of Coomassie brilliant blue staining solution. The blank control and standard groups were treated with 0.05 mL of distilled water and 0.05 mL of protein standard solution, respectively. After mixing and standing for 10 min, the spectrophotometer was zeroed with distilled water and absorbance was read at 595 nm. The following formula was used for calculation:

Soluble protein content(g/L) = (A_Sample_ − A_Blank_)/(A_Standard_ − A_Blank_) × Standard concentration × N

Note: N is the dilution factor used during the assay.

#### 4.3.3. CAT Activity

For the measurement of CAT activity, the ammonium molybdate method was used. After centrifuging the homogenate at 3500 rpm for 10 min, 0.05 mL of the supernatant was added to 1 mL of reagent 1 (at 37 °C) and 0.1 mL of reagent 2 (at 37 °C), mixed immediately, and reacted precisely for 1 min at 37 °C. Then, 1 mL of reagent 3 and 0.1 mL of reagent 4 were added and mixed. The spectrophotometer was zeroed with distilled water and absorbance was read at a wavelength of 405 nm. In the control group, the homogenate was replaced with distilled water and a similar procedure was followed. The following formula was used for calculation:

Activity of CAT (U/mL) = (ΔA × 271)/(V_s_ × T)

Note: ΔA = A_control_ − A_sample_;

271 is the molar extinction coefficient, in L/(mol·cm);

V_s_: sample volume, 0.05 mL;

T: reaction time, 60 s.

#### 4.3.4. SOD Activity

Here, the hydroxylamine method was employed. After centrifugation of the homogenate at 3500 rpm for 10 min, 1.0 mL of reagent 1, 0.05 mL of algal liquid, 0.1 mL of reagent 2, 0.1 mL of reagent 3, and 0.1 mL of reagent 4 were added to the experimental tube and mixed thoroughly. After a 40-min water bath at 37 °C, 2 mL of color reagent was added, mixed, and left to stand for 10 min. The spectrophotometer was zeroed with distilled water, and absorbance was read at 550 nm. In the control group, the homogenate was replaced with distilled water and a similar procedure was followed. The following formula was used for calculation:

SOD activity (U/mL) = (A_control_ − A_sample_)/(A_Control_ × 50%) × V_t_/V_s_

Note: V_t_ = Total reaction volume (mL);

V_s_ = Sample volume (mL).

#### 4.3.5. Proteomics

After UV irradiation, *Navicula* sp. liquid was mixed and centrifuged until 3 mL sediment remained for proteomic analysis. Label-free quantitative proteomics technology was used to obtain differential proteomic expression analysis.

## 5. Conclusions

In the present work, integrated physiologic and proteomic analyses were carried out to illustrate the molecular mechanism of *Navicula* sp. in response to UV irradiation stress. We found that ultraviolet radiation can alter the contents of soluble proteins and chlorophyll and increase the activity of antioxidation enzymes such as CAT in *Navicula* sp. cells. Differential protein GO analysis found that UV irradiation can activate the antioxidant system of *Navicula* sp. and further impact photosynthesis by affecting the photoreaction and chlorophyll synthesis of *Navicula* sp. The most significant differences in KEGG pathway analysis were also associated with those related to photosynthesis. The above results indicate that *Navicula* sp. has good UV radiation resistance ability by regulating its photosynthetic pigment content, photosynthetic activity, and antioxidant system, making it a potential candidate for the future development of space ecosystems.

## Figures and Tables

**Table 1:** Formula of CSI culture medium.

Number	Component	Concentration	Content
1	Ca(NO_3_)_2_·4H_2_O	15 g/100 mL dH_2_O	1 mL/L
2	KNO_3_	10 g/100 mL dH_2_O	1 mL/L
3	MgSO_4_·7H_2_O	4 g/100 mL dH_2_O	1 mL/L
4	β-Na_2_ glycerophosphate·5H_2_O	2.5 g/100 mL dH_2_O	1 mL/L
5	Vitamin B_12_		0.1 µg/L
6	Biotin		0.1 µg/L
7	Thiamine HCl		10 µg/L
8	HEPES		0.5 g/L
9	Na_2_SiO_3_·9H_2_O		0.1 g/L
10	Soil extract *		30 mL/L
11 (PIV)	Na_2_EDTA	0.75 g/L dH_2_O	6 mL/L
	MnCl_2_·4H_2_O	0.041 g/L dH_2_O	
	ZnCl_2_·7H_2_O	0.005 g/L dH_2_O	
	Na_2_MoO_4_·2H_2_O	0.004 g/L dH_2_O	
	FeCl_3_·6H_2_O	0.097 g/L dH_2_O	
	CoCl_2_·6H_2_O	0.002 g/L dH_2_O	

* Soil extract.

## Data Availability

The data that support the findings of this study are available from the corresponding author upon reasonable request.
